# Sleep Problems among Disaster Victims: A Long-Term Survey on the Life Changes of Disaster Victims in Korea

**DOI:** 10.3390/ijerph18063294

**Published:** 2021-03-23

**Authors:** Yujeong Kim, Haeyoung Lee

**Affiliations:** 1 College of Nursing, Research Institute of Nursing Science, Kyungpook National University, Daegu 41944, Korea; yujeongkim@knu.ac.kr; 2 Red Cross College of Nursing, Chung-Ang University, Seoul 06974, Korea

**Keywords:** sleep, disasters, natural disasters, posttraumatic stress disorder

## Abstract

This study aimed to assess sleep problems and their underlying factors in victims of disasters such as typhoons, heavy rains, fires, and earthquakes. Data from the long-term survey on life changes among disaster victims in 2019 obtained by the National Disaster Management Research Institute were used. The study included 1358 victims of natural and social disasters in Korea between 2012 and 2018. Sleep problems were assessed using a survey on subjective sleep quality and the use of sleeping medication. The data were analyzed using the chi-square test, *t*-test, and binominal logistic regression. The results showed that the factors affecting subjective sleep quality included disaster type, time elapsed after disaster, subjective health status, depression, and posttraumatic stress disorder. The factors affecting sleep medication use included age, time elapsed after disaster, and posttraumatic stress disorder. Therefore, interventions and support systems should be provided to prevent the prolongation of sleep problems.

## 1. Introduction

The incidence and severity of major disasters such as earthquakes, floods, and tsunamis have increased worldwide, threatening the health and well-being of individuals, families, and communities [1,2]. Disasters threaten lives, cause economic losses, and may worsen mental and physical health [3]. Posttraumatic stress disorder (PTSD) is the most common problem experienced by disaster victims. Those who get PTSD may also experience posttraumatic stress sequelae such as physical symptoms, including body pain, lower gastrointestinal disorder, skin disease, musculoskeletal disease, insomnia and fatigue, sleep disorder, major depression, anxiety disorder, alcohol use disorder, and suicide [4,5].

Problems with the quality of sleep are among the key characteristics of PTSD [6]. Unlike in the past when sleep quality problems were regarded as secondary symptoms of PTSD [7,8], sleep quality problems (e.g., rapid eye movement sleep disturbance, short- and long-term sleep impairment, insomnia, nightmares, etc.) are now emphasized as a core feature of PTSD [9,10,11]. There have been many studies on sleep quality problems among PTSD patients, and sleep quality problems are markers that reflect the severity of PTSD and predict the occurrence of PTSD [12,13,14,15]. Therefore, suppressing sleep quality problems is essential for alleviating PTSD among disaster survivors [16,17,18]. In particular, Miller et al. [11] showed that sleep disturbance after traumatic events could aggravate PTSD, further suggesting the importance of preventing sleep disturbance.

Sleep disturbance observed after a disaster is related to several demographic and environmental factors [19]. In previous studies, sleep disturbance was found to be more frequent in older female patients [20], and sleep duration (less than 5 h and more than 9 h) was related to low levels of education, unemployment, physical illness, anxiety disorder, and social phobia [21]. In particular, old age, widowed/divorced/separated marital status, and high physical activity levels and pain/discomfort were related to short sleep duration (less than 5 h). In studies on prolonged sleep difficulties due to disasters, social support, especially emotional social support in its three dimensions, was significantly related to prolonged sleep difficulties [22].

As disaster mental health is heavily affected by socio-cultural influences [23], survivors may react differently to the same disaster. Therefore, it is necessary to consider the characteristics of disaster victims, including their socio-cultural differences and their experiences before and after the disaster, to understand the victims’ health problems [24]. In addition, recent research reports that studies on sleep disturbance in not only high-risk groups (women, the elderly, and those with other mental health problems) but also in young and healthy adults are necessary to establish psychosocial and medical support for this demographic [25].

Therefore, this study assessed sleep problems and related factors among disaster victims to provide primary data for intervention programs that can help the victims in disaster response and recovery processes in the future.

## 2. Materials and Methods

### 2.1. Design

This is a cross-sectional research study designed to identify factors that affect sleep problems in adult disaster victims in South Korea.

### 2.2. Participants

In this study, data from the 4th long-term survey on the change of life of disaster victims in 2019 by the National Disaster Management Research Institute (NDMI) were used [26]. The survey was conducted among Korean victims of natural disasters, such as typhoons, heavy rains, earthquakes, and social disasters, including fires, from 2012 to 2018. A follow-up investigation to assess changes in disaster victims’ lives started with the first-panel survey in 2016 and ended with the fourth survey in 2019. A systematic extraction method that considered the participants’ gender, age, and residence was, after determining the size of the quota sample for each disaster type, used to extract the survey panel participants. In 2019, 2300 participants were surveyed, and in our study, a total of 1358 participants were included in the final analysis, excluding 66 and 876 participants under the age of 19 and over the age of 65, respectively. Elderly people over 65 years are more likely to have sleep problems than adults below this age. In addition, the elderly were reported to have far more factors affecting sleep problems than younger adults, including aging. Therefore, this study included only adults younger than 65 years in order to focus on sleep problems caused by disasters.

### 2.3. Measures

#### 2.3.1. General Characteristics

General characteristics such as gender, age, marital status, education level, household type, average monthly income, smoking, and alcohol abuse were assessed. Marital status was divided into married and unmarried/divorced/separated. Education level was classified as middle school graduate or lower and high school graduate and above, and the household type was divided into single-person and multi-person households. Using the average monthly total income of all family members living with the participant, average monthly income was categorized into less than KRW 3 million and over KRW 3 million. Smoking was assessed as yes or no, and alcohol abuse was evaluated using the alcohol use disorder identification test in Korea (AUDIT-K). The AUDIT-K consists of 10 items evaluated on a 5-point Likert scale from 0 points (“not at all”) to 4 points (“almost every time”). The total score ranges from 0 to 40, and a score of 15 or higher is classified as alcohol abuse [27].

#### 2.3.2. Disaster-Related Characteristics

Disaster-related characteristics included items related to disaster type, post-disaster period, and injury/illness damage. Disaster types referred to natural disasters, including typhoons, heavy rains, fires, and earthquakes. Time elapsed after a disaster was calculated as the period from the time of disaster until the 2019 survey, and was categorized as 2 years or less, 3–5 years, and 6–7 years. Injury/illness damage was assessed as yes or no using the item “Did you suffer injury or illness damage from the disaster?”

#### 2.3.3. Subjective Health Status.

Subjective health status was evaluated using a single item assessing current overall health status. The item was evaluated on a 7-point Likert scale from 1 point (“very bad”) to 4 points (“normal”) and 7 points (“very good”), and the higher the score, the greater the subjective health status. A subjective health status score of 1–3, 4, and 5–7 was assessed as bad, normal, and good, respectively.

#### 2.3.4. Psychological Factors

Psychological factors included depression, anxiety, PTSD, and resilience. Depression was assessed using the Patient Health Questionnaire-9 (PHQ-9) [28], which was translated into Korean and assessed for validity [29]. The PHQ-9 consists of 9 items on depressive symptoms in the last two weeks, evaluated on a 4-point Likert scale from 0 points (“not at all”) to 3 points (“nearly every day”). The total score was then calculated by summing the scores of all items, and the higher the score, the higher the severity of the depression. At the time of development and in our study, the Cronbach’s α of the tool was 0.95 and 0.92, respectively.

Anxiety was measured using the Generalized Anxiety Disorder-7 (GAD-7) scale [30] translated into Korean by the developers. The GAD-7 scale consists of 7 items on anxiety symptoms over the last two weeks, evaluated on a 4-point Likert scale from 0 points (“not at all”) to 3 points (“nearly every day”). The total score was calculated by summing the scores of all items, and the higher the score, the higher the level of anxiety. At the time of development and in our study, the Cronbach’s α of the tool was 0.92 and 0.93, respectively.

Similarly, PTSD was measured using the Korean translated version of the revised impact of event scale (IES-R) of Weiss and Marmar [31], and its modified version, IES-R-K [32]. The IES-R-K consists of 22 items on emotions and thoughts related to trauma events in the past week, evaluated on a 5-point Likert scale from 0 points (“not at all”) to 4 points (“very often”). The total score is then calculated by summing the scores of all items, and the higher the score, the higher the severity of PTSD. At the time of development and in our study, the Cronbach’s α of the tool was 0.83 and 0.98, respectively.

#### 2.3.5. Social Factors

Social factors included social support and social maladjustment. Social support was measured using the social support tool developed by Park [33] and modified to 12 items in four sub-areas (emotional support, evaluative support, information support, and material support) by the NDMI. The items were evaluated on a 5-point Likert scale from 1 point (“not at all”) to 5 points (“very much”), and the higher the score, the higher the after-disaster social support. At the time of development and in our study, the Cronbach’s α of the tool was 0.94 and 0.95, respectively.

Social maladjustment was assessed using the work and social adjustment scale developed by Mundt, Marks, Shear, and Greist [34], which consists of 5 items to assess the disaster’s effects on work ability, interpersonal relationships, and leisure activities. The items were evaluated on a 5-point Likert scale from 0 points (“not at all”) to 4 points (“very much”). The higher the score, the higher the social maladaptation. At the time of development and in our study, the Cronbach’s α of the tool was 0.78–0.94 and 0.97, respectively.

#### 2.3.6. Sleep Problems

Sleep problems were assessed using items on subjective sleep quality and use of sleeping medication from the Pittsburgh Sleep Quality Index (PSQI) [35]. Subjective sleep quality was assessed using the item “how is your overall quality of sleep over the last month?” which was evaluated on a 4-point Likert scale from 1 point (“not very good”) to 4 points (“very good”); “not very good” and “not good” were classified as bad, while “fairly good” and “very good” were classified as “good.”

Use of sleeping medication was assessed using the item “how often did you take medications (including both drugs prescribed from hospitals and purchased from pharmacies)?”, which was evaluated on a 4-point Likert scale from 1 point (“not at all”) to 4 points (“very often (nearly every day when necessary)”). In the analysis, there were no significant differences in variables such as depression, anxiety, PTSD, social support, and social maladjustment between the group taking sleep medication 1–2 times a month, the group taking it 1–2 times a week, and the group taking it daily. As such, “not at all” was classified as “not taken,” and “sometimes (once or twice a month when necessary)”, “occasionally (once or twice a week when necessary)”, and “very often (nearly every day when necessary)” were classified as “taken”.

### 2.4. Data Collection and Ethical Considerations

Researchers of the NDMI visited individual households and collected data through face-to-face interviews. After the NDMI’s approval, data were obtained in an anonymous format. Analysis of the data was performed after receiving Institutional Review Board (IRB) approval (no. KNU-2021-0009).

### 2.5. Data Analysis

The participants’ characteristics were analyzed using descriptive statistics, and the differences in sleep problems according to the characteristics of the participants were analyzed by the chi-square test and *t*-test. Using the SPSS IBM 25 program (IBM, Armonk, NY, USA), binomial logistic regression was performed to analyze factors that affected sleep problems.

## 3. Results

### 3.1. Characteristics of the Participants

Among the 1358 participants, 626 (46.1%) and 732 (53.9%) were male and female, respectively, and the mean age of the participants was 49.57 ± 12.30 years. In total, 974 (71.7%), 1083 (79.7%) and 1284 (94.6%) participants were married, high school graduates, and lived in a multi-person household, respectively. Of the participants, 687 (50.6%) had an average monthly income of more than KRW 3 million, while 238 (17.5%) and 35 (2.6%) were smokers and alcohol abusers, respectively. For disaster type, 413 (30.4%), 397 (29.2%), 351 (25.8%), and 197 (14.5%) participants suffered from heavy rain, earthquake, typhoon, and fire, respectively. The time elapsed after the disaster was less than 2 years and 3–5 years for 660 (48.6%) and 529 (39.0%) participants, respectively. The majority of the participants (1282 (94.4%)) did not report injury or illness caused by the disaster, and most of them (853 (62.8%)) had a good subjective health status. The mean score for depression, anxiety, and PTSD was 2.74 ± 4.05, 2.07 ± 3.19, and 13.78 ± 14.64 points, respectively. The mean score for social support and social maladaptation was 41.46 ± 7.54 and 7.84 ± 4.22 points (Table 1).

### 3.2. Differences in Subjective Sleep Quality According to the Characteristics of the Participants

Among 1358 participants, 313 (23.0%) had poor subjective sleep quality. Those with the characteristics of being female, 40–64 years old, married, an under middle-school graduate, living in a single-person household, having an average monthly income of less than KRW 3 million, being a non-smoker and a victim of heavy rain and fire, and having a disaster experience within the last 2 or 6–7 years had a lower subjective sleep quality. Additionally, the higher the severity of depression, anxiety, PTSD, or social maladjustment, and the lower the social support, the lower the subjective sleep quality. There was no significant difference according to other variables (Table 2).

### 3.3. Differences in Sleep Medication Use According to the Characteristics of the Participants

In this study, 141 (10.4%) participants used sleep medications. Participants over the age of 40, who were married under middle-school graduates, with an average monthly income less than KRW 3 million, and had a disaster experience within the last 2 years and a low subjective health status, displayed sleep medication use. Additionally, the higher the severity of the depression, anxiety, PTSD, or social maladjustment, and the lower the social support, the higher the use of sleep medication. There was no significant difference according to the other variables (Table 2).

### 3.4. Factors That Affect Subjective Sleep Quality

The gender, age, marital status, education, type of household, average monthly income and smoking status, which were general characteristic variables with significant differences in subjective sleep quality, were used as control variables. Disaster type, time elapsed after disaster, subjective health status, depression, anxiety, PTSD, social support, and social maladjustment were included as independent variables. The categorical variables were processed as dummy variables to analyze factors that affected the subjective sleep quality. Subjective sleep quality was classified as “good” (0) and “bad” (1). The regression model had Nagelkerke R^2^ = 0.37 with an explanatory power of 37.0%. The classification accuracy was 83.5%, which was satisfactory. The Hosmer–Lemeshow goodness-of-fit test showed *p* = 0.915, suggesting that the model was statistically suitable.

The subjective sleep quality odds ratio increased 2.75 and 1.66 times when the disaster type was a typhoon and heavy rain, respectively, compared to an earthquake, suggesting that typhoons and heavy rains led to poor subjective sleep quality compared to earthquakes. The subjective sleep quality odds ratio increased 0.31 times when the time elapsed after a disaster was 3–5 years compared to within 2 years, suggesting that the subjective sleep quality was better when the elapsed time after a disaster was 3–5 years. The subjective sleep quality odds ratio increased 1.92 and 3.49 times with a normal and low subjective health status, respectively, compared to high subjective health status, suggesting that the subjective sleep quality was worse in those with normal and low subjective health status. Additionally, every one-point increase in the PTSD scores increased the subjective sleep quality odds ratio by 1.03, suggesting that the higher the severity of PTSD, the lower the subjective sleep quality (Table 3).

### 3.5. Factors That Affect Sleep Medication Use

Variables with significant differences in sleep medication use were included as independent variables, and categorical variables were processed as dummy variables in analyzing factors that affected sleep medication use. The age, marital status, education, type of household, and average monthly income, which were general characteristic variables related to significant differences in sleep medication use, were put into control variables. Use and no use of sleep medications were assigned 1 and 0, respectively. The regression model had Nagelkerke R^2^ = 0.21 with an explanatory power of 20.8%. The classification accuracy was 89.2%, which was satisfactory. The Hosmer–Lemeshow goodness-of-fit test showed *p* = 0.571, suggesting that the model was statistically suitable.

Every one-year increase in age increased the odds ratio of sleep medication use by 1.03 times, suggesting that the sleep medication use increased. The sleep medication use odds ratio increased 0.49 times when the time elapsed after a disaster was 3–5 years compared to within 2 years, suggesting that sleep medication use decreased. Additionally, every one-point increase in the PTSD scores increased the sleep medication use odds ratio by 1.03, suggesting that the higher the severity of PTSD, the higher the sleep medication use (Table 4).

## 4. Discussion

In this study, the factors that affect sleep quality and sleep medication use among victims of disasters, such as typhoons, heavy rains, fires, and earthquakes, were analyzed. The main points of discussion based on the results of this study are as follows.

In our study, 23% and 10.4% of the participants showed poor subjective sleep quality and used sleep medications, respectively. As there are differences in the characteristics of the participants and methods used for assessing sleep problems, direct comparison is difficult; however, the proportion of participants with poor sleep quality is higher than the 6.9% [22] and 15% [36] observed in studies that assessed the prevalence of sleep difficulties in survivors of the Great East Japan Earthquake. In a recent study, considering that the prevalence of insomnia in Korean adults was 1.58% (20 s) to 10.28% (60 s) [37], this suggests that the participants in this study had many sleep problems.

An analysis of subjective sleep quality according to the characteristics of the participants showed that females over the age of 40 who were married, graduated from middle school or lower, had an average monthly income of less than KRW 3 million, were non-smokers, were victims of heavy rain or fire, and had experienced a disaster within the last 2 or 6–7 years had lower subjective sleep quality. In previous studies, the demographic characteristics shown to be related to duration of sleep difficulties were being female [21,37], older age [37], low income [21,38,39,40], less education [21,38,39], unemployment [38,41], and physical illness [21]. The findings in our study, therefore, corroborate the results of previous studies.

Additionally, the higher the severity of the depression, anxiety, PTSD, or social maladjustment, and the lower the social support, the lower the subjective sleep quality. This corroborates the results of a previous study, in which negative psychosocial factors, including financial strain, social isolation, low emotional support, negative social interactions, and psychological distress, were found to be related to sleep difficulties [42]. Social support also greatly affected sleep difficulties in a previous study of victims of the Great East Japan Earthquake [22]. Social support is a function of a social network and reflects the relationship between the family, group, community members, and the participants. Our finding that emotional support had the most significant effects on subjective sleep quality suggests that the act of sharing disaster experiences, emotional empathy, and support within a social network can positively affect sleep difficulties.

A binary logistic regression analysis showed that marital status, household type, disaster type, time elapsed after a disaster, subjective health status, depression, and PTSD affect the sleep quality of disaster victims. In a previous study, women and older people were reported to be mentally vulnerable to disaster-like situations [25], and Onose et al. [43] reported that women were more susceptible to PTSD. In our study, PTSD was a significant variable affecting sleep quality; however, gender was, surprisingly, not a significant variable in the regression analysis. One of the main factors associated with stress vulnerability is biological sex [44], and stress is associated with the incidence and severity of several psychiatric disorders, including PTSD and depression, that occur more often in women than in men [44]. Therefore, a sex-based approach to sleep disturbance and mental health management to resolve sleep disturbance would be important in reducing the PTSD resulting from disasters [25].

The subjective sleep quality odds ratio increased by 2.75 and 1.66 times when the disaster type was typhoon and heavy rain, respectively, compared to when it was an earthquake, suggesting that typhoon and heavy rain led to poor subjective sleep quality compared to earthquakes. In Korea, heavy rains caused by typhoons are frequent, and earthquakes are relatively rare. It is thought that a more detailed analysis of the difference in sleep quality according to the disaster type should also include the scale of damage caused by the disaster and the level of recovery.

We also observed that the subjective sleep quality odds ratio increased 0.31 times when the time elapsed after a disaster was 3–5 years compared to within 2 years, suggesting that the subjective sleep quality was better when the time elapsed after a disaster was 3–5 years. It is possible that the victims gradually recover from the trauma and that the quality of sleep improves simultaneously [45]. However, several participants still had a low quality of sleep even after 6–7 years since their disaster experience. Therefore, it would be necessary to continuously pay attention to, and manage, PTSD and other sleep difficulties resulting from disasters to prevent chronic difficulties among the victims.

The subjective sleep quality odds ratio increased 1.92 and 3.49 times in normal and low subjective health statuses, respectively, compared to high subjective health status, suggesting that the subjective sleep quality was worse in those with normal and low subjective health status. The participants’ health problems were not assessed in detail in this study. However, since physical illness is highly related to sleep difficulties [21], it would be important to rapidly identify and treat physical and mental health problems in disaster victims in order to manage sleep quality.

In this study, we also observed that participants over the age of 40 who were married, graduated from middle school or lower, had an average monthly income of less than KRW 3 million, had experienced a disaster within the last 2 years, and had a low subjective health status used sleep medications. Additionally, the higher the severity of depression, anxiety, PTSD and social maladjustment, and the lower the social support, the higher the sleep medication use. Our analysis also shows that age, time elapsed after a disaster, and PTSD significantly affect sleep medication use. Every one-year increase in age increased the odds ratio of sleep medication use 1.03 times, suggesting that the sleep medication use increased. The quality of sleep decreases as age increases; thus, it is thought that more people would require sleep medications to manage sleep difficulties. The sleep medication use odds ratio increased 0.49 times when the time elapsed after a disaster was 3–5 years compared to within 2 years, suggesting that sleep medication use decreased. The risk of sleep medication use should decrease as the time elapsed after disaster increases. However, there are individuals who experienced a disaster 6–7 years ago and still require sleep medications, and it is worth noting that the proportion of those who need sleep medication is greater here than in those who experienced a disaster 3–5 years ago. Considering that PTSD is also a factor that affects sleep medication use, it would be important to develop proactive measures that can help disaster victims recover from PTSD quickly. Additionally, it would be necessary to manage it with continuous monitoring to prevent prolonged sleep difficulties.

In a study of the victims of the 2011 Great East Japan Earthquake, financial hardship carried a high risk of lowering sleep duration and quality, and significantly increased the risk of inadequate sleep and insomnia. Furthermore, home destruction was a significant predictor of sleep medication use [19]. As the damage caused by a disaster is high, the resulting trauma is likely more severe, which may predictably increase sleep medication use. On the other hand, for those with relatively insignificant damage resulting from the disaster, such as in earthquake victims who did not lose their home and did not have to stay in shelters, social support, especially emotional support, was significantly more related to sleep difficulties than the nonmodifiable or hardly modifiable consequences of disasters (e.g., housing damage, changes in family structure) [22]. Thus, it would be necessary to select an appropriate intervention plan considering that factors that influence sleep problems may vary depending on the level of damage caused by the disaster.

The recommended sleep duration per day is approximately 7–8 h, and more or less sleep is related to various health problems such as high blood pressure, cardiovascular disease, diabetes, obesity [46,47], and mental health problems, including depression [48]. Preventing and managing the sleep disorders observed after a disaster and the resulting various health problems would be helpful to not only the recovery of individual disaster victims, but also the recovery of communities affected by disasters [36]. Therefore, more attention and active measures would be necessary.

The limitations of this study are as follows. First, the subjective sleep quality of the participants was evaluated and analyzed as a binary variable. Sleep medication use was also assessed depending on the response of the participants. Therefore, further studies are required to assess objective sleep quality and verify the relationship between sleep quality and PTSD caused by disasters. Moreover, it is also necessary to conduct a study that assesses sleep medication use based on the type and dose of actual drugs rather than participants’ responses. Second, as this was a cross-sectional research study, it was not possible to verify the causal relationship of factors that affect sleep quality. A longitudinal observation of factors that affect the sleep of disaster victims and a chronological analysis of the accumulated data is required. Third, related variables, such as the victims’ sleep difficulties before the disaster, traumatic events, the scale of damage caused by the disaster, and the recovery level, were not considered in our study. It would be necessary to analyze related factors in the consideration of the effects of these variables on sleep quality and the severity of PTSD induced by disasters. Nevertheless, this study is meaningful in that it is based on panel data from disaster victims in Korea, and assessed factors that affect sleep quality and sleep medication use.

## 5. Conclusions

It is difficult to predict the time and place of natural disasters, and anyone can be a victim of disasters. Therefore, it is necessary for everyone to be interested and actively facilitate victims’ quick recovery during disasters. Attention to disaster-related sleep quality problems is essential to alleviating PTSD among survivors. Our study observes that the risk of low sleep quality and sleep medication use is greater among the socially underprivileged who have low education levels, low incomes, high social maladjustment, and low social support. Moreover, the time elapsed after disaster and PTSD were highly related to sleep quality and sleep medication use. Therefore, more focus on preventing and mitigating sleep difficulties in the event of disaster-related damages would be important. In particular, rapid therapeutic interventions should be provided to prevent prolonged sleep difficulties following disasters, and measures for continuous interest and support systems rather than short-term support need to be developed.

## Figures and Tables

**Table 1:** Participants’ characteristics (*N* = 1358).

Variables	Category	*n* (%), M±SD
Gender	Male	626(46.1%)
	Female	732(53.9%)
Age	19–39	324(23.9%)
	40–64	1034(76.1%)
	Total	49.57 ± 12.30
Marital status	Single/divorced/widow	384(28.3%)
	Married	974(71.7%)
Education	≤Middle school	275(20.3%)
	≥High school	1083(79.7%)
Type of household	Single-person household	74(5.4%)
	Multi-person household	1284(94.6%)
Average monthly income	<KRW 3 million	671(49.4%)
	≥KRW 3 million	687(50.6%)
Smoking	No	1120(82.5%)
	Yes	238(17.5%)
Alcohol abuse	No	1323(97.4%)
	Yes	35(2.6%)
Disaster type	Typhoon	351(25.8%)
	Heavy rain	413(30.4%)
	Fire	197(14.5%)
	Earthquake	397(29.2%)
Time elapsed after disaster (year)	≤2	660(48.6%)
	3–5	529(39.0%)
	6–7	169(12.4%)
Injury/illness damage	No	1282(94.4%)
	Yes	76(5.6%)
Subjective health status	Low	163(12.0%)
	Moderate	342(25.2%)
	High	853(62.8%)
Depression (points)		2.74 ± 4.05
Anxiety (points)		2.07 ± 3.19
PTSD (points)		13.78 ± 14.64
Social support (points)		41.46 ± 7.54
Social maladjustment (points)		7.84 ± 4.22

Note: PTSD, posttraumatic stress disorder.

**Table 2:** Differences in subjective sleep quality and sleep medication use according to participants’ characteristics (*N* = 1358).

Variables	Category	Subjective Sleep Quality			Sleep Medication Use		
		Good (*n* = 1045)	Bad (*n* = 313)	x^2^, t (*p*)	No (*n* = 1217)	Yes (*n* = 141)	x^2^, t (*p*)
Gender	Male	507(81.0%)	119(19.0%)	10.68 (0.001)	568(90.7%)	58(9.3%)	1.56 (0.212)
	Female	538(73.5%)	194(26.5%)		649(88.7%)	83(11.3%)	
Age	19–39	279(86.1%)	45(13.9%)	20.13 (<0.001)	308(95.1%)	16(4.9%)	13.56 (<0.001)
	40–64	766(74.1%)	268(25.9%)		909(87.9%)	125(12.1%)	
Marital status	Single/divorced/widow	314(81.8%)	70(18.2%)	7.01 (0.008)	355(92.4%)	29(7.6%)	4.61 (0.032)
	Married	731(75.1%)	243(24.9%)		862(88.5%)	112(11.5%)	
Education	≤Middle school	192(69.8%)	83(30.2%)	9.89 (0.002)	224(81.5%)	51(18.5%)	24.69 (<0.001)
	≥High school	853(78.8%)	230(21.2%)		993(91.7%)	90(8.3%)	
Type of household	Single-person household	43(58.1%)	31(41.9%)	15.67 (<0.001)	65(87.8%)	9(12.2%)	2.67 (0.606)
	Multi-person household	1002(78.0%)	282(22.0%)		1152(89.7%)	132(10.3%)	
Average monthly income (KRW millions)	<3	465(69.3%)	206(30.7%)	43.79 (<0.001)	572(85.2%)	99(14.8%)	27.24 (<0.001)
	≥3	580(84.4%)	107(15.6%)		645(93.9%)	42(6.1%)	
Smoking	No	850(75.9%)	270(24.1%)	4.04 (0.045)	1002(89.5%)	118(10.5%)	0.16 (0.689)
	Yes	195(81.9%)	43(18.1%)		215(90.3%)	23(9.7%)	
Alcohol abuse	No	1021(77.2%)	302(22.8%)	1.42 (0.233)	1188(89.8%)	135(10.2%)	1.76 (0.184)
	Yes	24(68.8%)	11(31.4%)		29(82.9%)	6(17.1%)	
Disaster type	Typhoon	282(80.3%)	69(19.7%)	11.86 (0.008)	316(90.0%)	35(10.0%)	2.04 (0.565)
	Heavy rain	298(72.2%)	115(27.8%)		376(91.0%)	37(9.0%)	
	Fire	145(73.6%)	52(26.4%)		173(87.8%)	24(12.2%)	
	Earthquake	320(80.6%)	77(19.4%)		352(88.7%)	45(11.3%)	
Time elapsed after disaster(year)	≤2	479(72.6%)	181(27.4%)	26.64 (<0.001)	575(87.1%)	85(12.9%)	9.59 (0.008)
	3–5	446(84.3%)	83(15.7%)		490(92.6%)	39(7.4%)	
	6–7	120(71.0%)	49(29.0%)		152(89.9%)	17(10.1%)	
Injury/illness damage	No	52(68.4%)	24(31.6%)	3.30 (0.069)	1153(89.9%)	129(10.1%)	2.53 (0.112)
	Yes	993(77.5%)	289(22.5%)		64(84.2%)	12(15.8%)	
Subjective health status	Low	65(39.9%)	98(60.1%)	197.81 (<0.001)	124(76.1%)	39(23.9%)	44.91 (<0.001)
	Moderate	232(67.8%)	110(32.2%)		299(87.4%)	43(12.6%)	
	High	748(87.7%)	105(12.3%)		794(93.1%)	59(6.9%)	
Depression		1.78 ± 2.98	5.95 ± 5.29	−13.32 (<0.001)	2.38 ± 3.74	5.82 ± 5.15	−7.72 (<0.001)
Anxiety		1.39 ± 2.40	4.35 ± 4.25	−11.80 (<0.001)	1.78 ± 2.96	4.58 ± 3.90	−8.26 (<0.001)
PTSD		10.68 ± 12.04	24.12 ± 17.53	−12.70 (<0.001)	12.44 ± 13.90	25.36 ± 15.75	−10.30 (<0.001)
Social support		42.00 ± 7.46	39.66 ± 7.55	4.85 (<0.001)	41.72 ± 7.54	39.21 ± 7.15	3.94 (<0.001)
Social maladjustment		7.49 1 ± 4.01	9.00 ± 4.69	−5.17 (<0.001)	7.69 ± 4.10	9.09 ± 4.99	−3.19 (0.002)

**Table 3:** Factors associated with subjective sleep quality.

Variables		B	SE	Wald	OR	95% CI	*p*
Intercept		−2.67	0.68	15.44	0.07		<0.001
Gender (ref = male)		0.28	0.18	2.40	1.32	0.93–1.89	0.121
Age		0.01	0.01	0.81	1.01	0.99–1.03	0.369
Marital status (ref = Single/divorced/widow)		0.35	0.24	2.13	1.42	0.89–2.26	0.145
Education (ref = ≥High school)		−0.34	0.20	2.74	0.71	0.48–1.06	0.098
Type of household (ref = multi-person household)		0.69	0.36	3.75	2.00	0.99–4.04	0.053
Average monthly income (ref = ≥KRW 3 million)		0.26	0.17	2.36	1.29	0.93–1.80	0.124
Smoking (ref = no)		−0.00	0.24	0.00	1.00	0.62–1.60	0.986
Disaster type (ref = earthquake)	Typhoon	1.01	0.31	10.47	2.75	1.49–5.07	0.001
	Heavy rain	0.51	0.21	5.89	1.66	1.10–2.51	0.015
	Fire	0.00	0.28	0.00	1.00	0.58–1.73	0.989
Time elapsed after disaster (ref = ≤2 years)	3–5	−1.16	0.25	21.59	0.31	0.19–0.51	<0.001
	6–7	−0.54	0.32	2.84	0.59	0.31–1.09	0.092
Subjective health status (ref = high)	Low	0.65	0.18	13.44	1.92	1.35–2.71	<0.001
	Moderate	1.25	0.23	28.79	3.49	2.21–5.51	<0.001
Depression		0.14	0.03	20.23	1.15	1.08–1.22	<0.001
Anxiety		0.06	0.04	2.58	1.06	0.99–1.14	0.108
PTSD		0.03	0.01	16.87	1.03	1.02–1.04	<0.001
Social support		−0.02	0.01	1.94	0.99	0.96–1.01	0.164
Social maladjustment		−0.03	0.02	2.16	0.97	0.92–1.01	0.142

Subjective sleep quality: good = 0, bad = 1; OR, odds ratio; CI, confidence interval, ref.: Reference.

**Table 4:** Factors associated with sleep medication use.

Variables		B	SE	Wald	OR	95% CI	*p*
Intercept		−3.57	0.81	19.43	0.03		<0.001
Age		0.03	0.01	7.25	1.03	1.01–1.05	0.007
Marital status (ref = Single/divorced/widow)		0.11	0.26	0.17	1.11	0.67–1.83	0.681
Education (ref = ≥High school)		0.44	0.23	3.81	1.55	1.00–2.41	0.051
Average monthly income (ref = ≥ KRW 3 million)		0.36	0.22	2.77	1.43	0.94–2.19	0.096
Time elapsed after disaster (ref = ≤2 years)	3–5	−0.71	0.24	8.36	0.49	0.31–0.80	0.004
	6–7	-0.36	0.31	1.31	0.70	0.38–1.29	0.252
Subjective health status (ref = high)	Low	−0.05	0.23	0.04	0.95	0.60–1.51	0.840
	Moderate	0.15	0.28	0.29	1.16	0.67–2.02	0.593
Depression		0.03	0.03	0.86	1.03	0.97–1.10	0.353
Anxiety		0.08	0.04	3.33	1.08	0.99–1.17	0.068
PTSD		0.03	0.01	12.10	1.03	1.01–1.05	0.001
Social support		−0.03	0.01	3.38	0.98	0.95–1.00	0.066
Social maladjustment		−0.03	0.03	0.99	0.97	0.92–1.03	0.319

SE, standard error; OR, odds ratio; CI, confidence interval, ref.: Reference.

## Data Availability

The data presented in this study are available on request from the National Disaster Management Research Institute, Republic of Korea (http://www.ndmi.go.kr/research/research/view.jsp?DOC_ID=D0000015429 accessed on 20 February 2021).
